# An Improved Model of Heat-Induced Hyperalgesia—Repetitive Phasic Heat Pain Causing Primary Hyperalgesia to Heat and Secondary Hyperalgesia to Pinprick and Light Touch

**DOI:** 10.1371/journal.pone.0099507

**Published:** 2014-06-09

**Authors:** Tim P. Jürgens, Alexander Sawatzki, Florian Henrich, Walter Magerl, Arne May

**Affiliations:** 1 Department of Systems Neuroscience, University Medical Centre Hamburg-Eppendorf, Hamburg, Germany; 2 Chair of Neurophysiology, Center of Biomedicine and Medical Technology, Medical Faculty Mannheim, University Heidelberg, Mannheim, Germany; University of Texas at Dallas, United States of America

## Abstract

This study tested a modified experimental model of heat-induced hyperalgesia, which improves the efficacy to induce primary and secondary hyperalgesia and the efficacy-to-safety ratio reducing the risk of tissue damage seen in other heat pain models. Quantitative sensory testing was done in eighteen healthy volunteers before and after repetitive heat pain stimuli (60 stimuli of 48°C for 6 s) to assess the impact of repetitive heat on somatosensory function in conditioned skin (primary hyperalgesia area) and in adjacent skin (secondary hyperalgesia area) as compared to an unconditioned mirror image control site. Additionally, areas of flare and secondary hyperalgesia were mapped, and time course of hyperalgesia determined. After repetitive heat pain conditioning we found significant primary hyperalgesia to heat, and primary and secondary hyperalgesia to pinprick and to light touch (dynamic mechanical allodynia). Acetaminophen (800 mg) reduced pain to heat or pinpricks only marginally by 11% and 8%, respectively (n.s.), and had no effect on heat hyperalgesia. In contrast, the areas of flare (−31%) and in particular of secondary hyperalgesia (−59%) as well as the magnitude of hyperalgesia (−59%) were significantly reduced (all p<0.001). Thus, repetitive heat pain induces significant peripheral sensitization (primary hyperalgesia to heat) and central sensitization (punctate hyperalgesia and dynamic mechanical allodynia). These findings are relevant to further studies using this model of experimental heat pain as it combines pronounced peripheral and central sensitization, which makes a convenient model for combined pharmacological testing of analgesia and anti-hyperalgesia mechanisms related to thermal and mechanical input.

## Introduction

In previous studies, our group developed a repetitive nociceptive heat stimulation paradigm, which resulted in acute and pronounced intra-session sensitization and simultaneously an sustained inter-session habituation lasting for weeks related to activation of endogenous pain control [1]–[3]. The stimulus protocol involved the administration of repetitive noxious heat stimuli using a thermode, which were delivered in 10 blocks of 6 brief stimuli with a temperature of 48°C each. This standardized protocol of repetitive heat pain (RHP) was administered daily for 8 consecutive days. So far, the peripheral and central mechanisms of homotopic intra-session sensitization to repetitive noxious thermal stimuli have not been characterized in detail. Primary heat hyperalgesia induced by a strong thermal stimulation in the stimulated skin area is predominantly caused by sensitization of primary afferent nociceptors as a mainly peripheral phenomenon [4]. Tonic administration of heat leading to mild skin burns (first degree burn) is known to induce hyperalgesia to punctate mechanical stimuli surrounding the site of primary hyperalgesia [5] similar to changes observed after intra- or epidermal application of capsaicin [6], or repetitive intra- or epidermal electrical stimulation [7]. The common molecular denominator in these models is a strong input in capsaicin-sensitive nociceptors bearing the TRPV1 receptor. Strong input in these mostly peptidergic nociceptive primary afferents causes central sensitization of spinal neurons to input from capsaicin-insensitive nociceptive A-delta fibres [8]–[11]. The dynamic range of inputs causing central sensitization is remarkably wide. Even sustained nociceptor activation by heat stimuli at levels sufficient to produce a flare, which implies the activation of these afferents but does not necessarily cause a conscious perception of pain, and even completely painless UVB irradiation, have been found to trigger secondary hyperalgesia [12], [13]. Since established burn injury models are not completely safe and sometimes induce manifest tissue injuries (second degree burn) [14], which makes mechanistic interpretations difficult, we strived to improve this method by a train repeated brief heat stimuli rather than sustained high level heat as has been used previously.

Specifically, we aimed to address the following objectives in our study:

1. Previously established models of primary and secondary hyperalgesia can be difficult to dose and some of these models may induce tissue damage such as UV-induced sunburn with longer lasting hyperpigmentation or thermally induced second degree burns with blistering which limits their use in sensitive skin areas such as the face. Thus, our main objective was to characterize the magnitude of primary and secondary hyperalgesia, the area of hyperalgesia and its time course induced by our model, and to assess its efficacy-to-safety ratio.

2. An additional objective (proof-of-concept trial) was to determine whether modulation by infusion of acetaminophen was comparable to previously established models of central sensitization.

## Methods

### Ethics statement

The study was approved by the local Ethics Committee (protocol number PV3504) of the Medical Chamber of Hamburg and conformed to the Declaration of Helsinki. All participants gave written informed consent.

### Study design

We conducted a prospective controlled single-centre study to investigate the effects of repetitive phasic heat pain in thermal and mechanical hyperalgesia using quantitative sensory testing (QST) [15] in healthy humans. All subjects provided written informed consent prior to inclusion into the study.

### Subjects

Participants were recruited among medical students of Hamburg University. Eighteen subjects (9 male and 9 female, mean age 27.6±3.4 years, range 22–36 years) were included into the study. Only right-handed subjects between 18 and 65 years of age were eligible. Exclusion criteria were: chronic pain, acute pain within the last four weeks, any long-term medication apart from an oral contraceptive, intake of analgesics or hepatotoxic drugs within the last 72 hours, pregnancy and lactation, alcohol or drug addiction, liver disease and relevant psychiatric, neurologic or other disease.

### Experimental design

**Figure 1:**
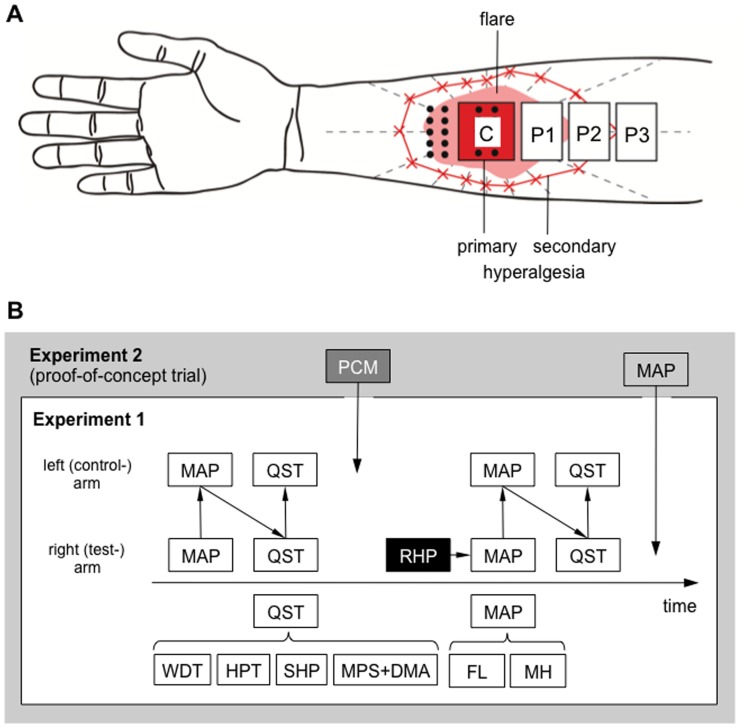
Experimental design. **A**: Schematic illustration of the tested areas in the main experiment (Experiment 1): Test zones and 16 vectors (dashed lines) were marked on the right and left volar forearm. At the central zone (C) on the right (i.e. test-) arm repetitive heat pain was applied inducing visible flare as well as primary and secondary hyperalgesia. Mechanical testing was done in the central zone and distal to it (dots), thermal testing in the central and peripheral zones (P1, P2 and P3). Areas of secondary hyperalgesia were mapped using a 256 mN von Frey hair. **B**: Test sequence: In the main experiment (Experiment 1), quantitative sensory testing (QST) started in the central zone and was repeated in the peripheral zones. Repetitive heat pain (RHP) was applied to the test arm only. In an additional experiment (Experiment 2) 800 mg acetaminophen (paracetamol) i.v. was administered to eight subjects 40 minutes before RHP. In this additional experiment, thermal testing was performed in the peripheral zone P1 only. DMA dynamic mechanical allodynia (pain to light touch), FL axon reflex erythema (flare), HPT heat pain threshold, MAP mapping of the areas of flare and secondary hyperalgesia, MH mechanical hyperalgesia, MPS rating of pain to punctate stimuli (calibrated pinpricks), PCM acetaminophen, SHP rating of suprathreshold heat pain, WDT warm detection threshold.

All participants underwent QST including warm detection thresholds, heat pain thresholds, ratings to suprathreshold heat stimuli and mechanical pain sensitivity to pinprick stimuli and brushing. Beforehand, the extent of secondary hyperalgesia and flare was mapped on both arms with the right being the test side and the left volar forearm the control side (see Figure 1 for details of central and peripheral test zones P1-3), as no relevant side differences have been found for QST variables in the same body region [16]. Then a previously established standardized heat pain paradigm [17], [18] with repetitive blocks of suprathreshold heat pain stimuli was applied to the test site. Thereafter, the areas of flare and secondary mechanical hyperalgesia were mapped first to ensure a constant time between thermal stimulation and flare assessment and to avoid further nociceptor activation by test stimuli. Then, all QST modalities were tested again. All assessments were done by the same investigator (AS) apart from the additional experiment on the time course of secondary hyperalgesia and flare (FH and WM).

### Heat pain paradigm

To assess subjective rating to heat pain we applied a repetitive heat pain paradigm which is published elsewhere. Heat stimuli were generated by a thermode with a Peltier element (contact area 30×30 mm, TSA-II NeuroSensoryAnalyser, Medoc Ltd. Advanced Medical Systems, Ramat Yishai, Israel). In short, the paradigm consists of 10 blocks with 6 noxious heat stimuli each (baseline temperature 32.0°C, target temperature 48.0°C, duration 6 s; temperature rise 10 °C/s). The thermode was strapped on the right volar forearm. Heat stimuli were triggered by an external computer with a custom-written application (Presentation 11, Neurobehavioral Systems Inc., Albany, CA, USA). After each block (inter-stimulus interval between blocks 30 s) subjects were asked to rate mean perceived pain on a visual analog rating scale (VAS) ranging from 0 (no pain) to 100 (most severe pain imaginable) using a computed device and stored for offline analysis.

### Quantitative sensory testing (Somatosensory test stimuli)

Testing of somatosensory perception was based on elements of a standardized test battery for quantitative sensory testing (QST) [15] which was developed as part of the German Research Network on Neuropathic Pain (DFNS). Before and after application of the heat pain paradigm skin and room temperature were measured in each zone on both the test and the control side. During the tests all subjects wore an opaque eye mask.

#### Pain ratings

Subjects rated the magnitude of pain to suprathreshold mechanical and thermal test stimuli on a numerical rating scale (NRS) ranging from 0 (non-painful) to 100 (most intense pain imaginable).

#### Thermal detection and pain thresholds

Thermal thresholds were determined by using a computerized thermode with Peltier elements (TSA-II NeuroSensory Analyzer, MEDOC, Israel) with a contact area of 16×16 mm (32°C baseline temperature, ramped stimuli with 1 °C/s for detection, 10 °C/s for pain thresholds). First, the thresholds of warm detection (WDT) were measured using the method of limits which requires the participants to indicate the first perception of warm and cold by pressing a button. This was followed by determination of heat pain threshold (HPT) using the same method of limits. Subjects were asked to abort the increasing thermal stimulus by pressing a button as soon as they perceived an additional burning, stabbing or piercing component in addition to the perception of warmth or heat. To avoid temporal summation of heat stimuli, HPT were separated by an interstimulus interval of 10 s. The mean threshold temperature of three consecutive measurements was calculated.

#### Suprathreshold heat stimuli

Suprathreshold heat pain stimuli (SHP) were applied on each zone (central and peripheral zone P1-3) by using the above thermal sensory testing device with a 16×16 mm contact area of the thermode head. Maintaining a constant temperature of 48 °C, the probe was attached manually to the test sites for 2 s each using a stopwatch to ensure exact timing. Then the probe was removed from the test site. The mean pain rating of three consecutive measurements was calculated. The number of heat stimuli was kept at an absolute minimum and the interstimulus interval was set to 10 s to prevent additional sensitization induced by temporal summation of thermal test stimuli. A small thermode head was chosen for SHP testing to reduce repeated testing of the same area within the larger central and peripheral testing sites and thus unwanted spatial summation.

#### Mechanical pain sensitivity for pinprick stimuli and dynamic mechanical allodynia for stroking light touch

Mechanical pain sensitivity (MPS) was assessed using custom-made weighted pinprick stimuli with fixed stimulus intensities (The Pinprick, MRC Systems, Heidelberg, Germany; 8, 16, 32, 64, 128, 256, 512 mN; flat contact area of 0.25 mm diameter) in order to define a stimulus-response function. These punctate stimuli were adequate to excite cutaneous nociceptors [19], [20]. Pain to light touch (dynamic mechanical allodynia, DMA) was tested by light stroking with a cotton wisp (3 mN), a cotton wool tip fixed to an elastic strip (100 mN) or a soft brush (Somedic SENSE Lab Brush, Sweden; 200–400 mN). Each of the seven intensities of pinpricks and of the three intensities of light stroking was applied five times in a randomized sequence, according to the DFNS protocol, in the central and peripheral zone P1 only due to temporal constraints.

Mechanical pain sensitivity was calculated as the geometric mean of all pain ratings for pinprick stimuli and allodynia was calculated as the geometric mean of all pain ratings after light touch stimuli.

#### Mapping and quantification of secondary hyperalgesia and flare

Mechanical detection of the area of secondary hyperalgesia developing in skin adjacent to the repetitive heat stimulation was assessed using a calibrated von Frey hair (Optihair_2_, Marstock Nervtest, Germany) that delivers a force of 256 mN (punctuate stimulus). The contact area of the von Frey hair with the skin was of uniform size and blunt shape (0.5 mm in diameter). Testing started outside the hyperalgesic area moving towards the centre (primary hyperalgesia) in 5-mm-steps on previously marked lines yielding 12 marks (see Figure 1a). The subjects were asked to indicate the point when the sensation of pressure/touch changed to a sensation of pain. The location was marked on the skin using a soft felt-tip pen as were those of the flare that developed around the stimulation site. The tagged points on the skin indicating hyperalgesia and the visible flare were transferred on an acetate sheet before (t_0_) and immediately after (t_1_) the pain paradigm. The sheets were then scanned (1∶1) and the areas of flare and hyperalgesia quantified using a computer-based system (Adobe Acrobat 9 Pro, San Jose, CA, USA).

Additionally, in a separate group of 8 subjects (S1-S8, 6 male and 2 female subjects; mean age 30±7 years, range 19–57 years) we characterized the time course of the heat-induced primary and secondary hyperalgesia immediately after, and at 1, 2, 4, 8, 12, and 24 h after RHP. To this end, we mapped the areas of secondary hyperalgesia and compared the pain ratings to a single force of pinprick of 256 mN in the primary and secondary hyperalgesia areas.

### Multidimensional pain questionnaire

The Hamburg Pain Adjective List (HSAL) is a validated 37 item list in German language [21]. Subjects are asked to rate different aspects of the perceived pain on a 7 point Likert scale. The questionnaire comprises 4 primary scales: pain suffering (PS), pain anxiety (PA), pain sharpness (PS) and pain rhythm (PR). Two secondary scales can be formed: AFFECTIVE (27 items) and SENSORY (16 items), which can be added to a TOTAL scale. It has been used in previous studies on pain [11] and has been designed specifically for detecting changes over time. The HSAL was completed by all 18 subjects. It was done thrice in each session: after the first, the fifth and the tenth (i.e. last) block of 6 noxious thermal stimuli.

### Pharmacological proof-of-concept trial: acetaminophen (paracetamol) administration

Eight of the 18 subjects underwent an additional single-session experiment (5 male, 3 female, mean age 26.5±2.4 years, range 22–30 years) in which the effects of acetaminophen on the formation of secondary hyperalgesia were tested. As the test session took place on different days and tests was separated from the main experiment by at least 6 weeks, the test sites remained identical for both sessions. All subjects received uniform instructions about the study design (double-blind, placebo-controlled), pharmacologic properties of acetaminophen and that they had a 50% chance of receiving saline to control for potential placebo effects. However, over a period of 10 minutes all participants of the second experiment received 800 mg acetaminophen (80 ml solution containing 10 mg/ml; Bristol-Myers-Squibb, Munich, Germany) was administered via an intravenous line into the cubital vein of the left arm. The dose was based on findings by Koppert et al., who showed that the effect of acetaminophen on secondary hyperalgesia by far outweighs its analgesic effects after electrically induced hyperalgesia [22],[23]. After the end of the infusion, 30 minutes (i.e. 40 minutes after starting the infusion) elapsed before the repetitive heat pain was applied again as maximum antihyperalgesic efficacy was found to occur 40 min after starting the infusion [22]. Ratings to repetitive heat pain and the area of secondary pinprick hyperalgesia and allodynia were assessed before administration of acetaminophen and after the repetitive heat pain. To keep this experiment as brief as possible, pain testing was only performed in central area and in one of the peripheral test areas (P1).

### Data evaluation and statistics

Heat pain thresholds, suprathreshold heat pain ratings, areas of flare and secondary hyperalgesia, and HSAL scores were analysed as raw data. Warm detection thresholds, which are usually not normally distributed were transformed into decadic logarithms in order to achieve secondary normal distribution [24]. Pain ratings to pinprick and to light touch returned a substantial number of zero pain ratings. Thus, a small constant (0.1) was added to all pain ratings (mechanical pain sensitivity and dynamic mechanical allodynia) to avoid loss of zero-values, and then the ratings were log-transformed (for theoretical background, see [25]).

Group differences were examined by means of an analysis of variance (ANOVA) for repeated measures. Mauchly's Test was used to detect violations of sphericity. In such cases, Greenhouse-Geisser correction was applied. For *post hoc* tests and pairwise comparisons, t-tests were used. Results with p<0.05 were regarded as significant. Statistical analysis was performed using SPSS 17 (SPSS Inc., Chicago, IL, USA).

## Results

### Repetitive heat pain (RHP)

**Figure 2:**
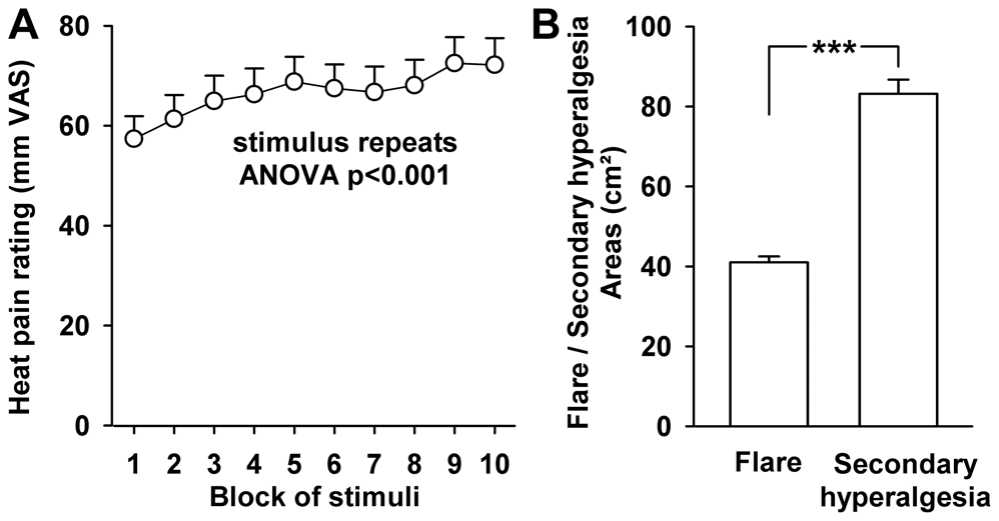
Pain ratings to repetitive 48°C heat stimuli, and heat-induced flare and secondary hyperalgesia. **A**: Pain ratings in 18 healthy volunteers upon repetitive heat pain stimulation (60 heat stimuli of 48°C with 6 s plateau repeated every 15 s). Rating on a visual analogue scale (VAS) ranging from 0 (no pain) to 100 mm (worst imaginable pain). The Figure depicts average pain ratings of blocks of six consecutive heat pain stimuli, each. **B**: Areas of heat-induced spreading erythema (flare) and of secondary hyperalgesia tested by stimulation with a 256 mN von Frey hair. *** p<0.001.

In 18 healthy volunteers, ratings to repetitive heat pain stimuli and heat-induced flare and secondary hyperalgesia were assessed (Figure 2). Mean pain intensity on the VAS was 57.4±4.5 in the first block, and heat pain ratings increased gradually from the first to the 10^th^ block (one-way RM-ANOVA: F_3.3, 55.7_ = 7.01, p<0.001. Pain ratings were significantly higher in any later test block compared to the first one (all at least p<0.05) amounting to a mean pain rating of 72.2±5.4 in the 10^th^ block, a 26% higher pain rating than in the first block (p<0.002) indicating significant sensitization in the heat-conditioned area (Figure 2A).

### Mapping of secondary hyperalgesia and flare

A visible flare developed during application of repetitive heat pain and continuously increased, extending beyond the contact area of the thermode head. Extension from the thermode was more pronounced in the proximal than distal direction of the forearm in all subjects. Secondary hyperalgesia and flare were absent at baseline testing. However, after the conditioning heat pain paradigm we identified significant areas of flare (mean area: 41.0±1.5 cm^2^; ANOVA: p<<0.001) and of secondary hyperalgesia (mean: 83.2±3.5 cm^2^; ANOVA: p<<0.001), which were characterized by their remarkable small inter-individual variation of magnitude (coefficients of variation of 0.19 and 0.17, respectively). The area of secondary hyperalgesia was significantly larger than the flare area (more than twice the size of the flare area; p<<0.001; Figure 2B).

The flare disappeared within one day and subjects reported no persistent effects beyond that day, such as erythema. Higher degrees of tissue damage (i.e. second degree burn injuries such as blistering) were never observed throughout the experiment.

### Quantitative sensory testing (QST) in the zones of primary and secondary hyperalgesia

Sensory changes were more comprehensively assessed quantitatively by thermal testing (warm detection threshold WDT, heat pain threshold HPT, and suprathreshold heat pain rating SHP) and mechanical testing (pain rating to pinprick stimuli MPS, and pain rating to tactile stimuli, i.e. dynamic mechanical allodynia DMA) in the heat pain-conditioned (“central”) and adjacent skin areas (“peripheral” P1–P3, see Figure 1). All QST parameters exhibited substantial reliability and a stable side-to-symmetry at baseline as evaluated by correlations (r = 0.64–0.86, all at least p<0.005) and absence of left-to-right difference (all p>0.30). A comprehensive overview of quantitative sensory testing results is given in Table 1.

**Table 1:** Mean values (mean) and 95% confidence intervals (CI) of the retransformed results.

		test side								control side							
		pre				post				pre				post			
		mean	CI			mean	CI			mean	CI			mean	CI		
**WDT [°C]**	C	**35,68**	34,81	-	36,58	**38,30**	37,30	-	39,32	**35,65**	34,86	-	36,47	**36,04**	35,07	-	37,03
	P1	**35,45**	34,82	-	36,08	**36,31**	35,39	-	37,26	**35,03**	34,56	-	35,50	**35,15**	34,75	-	35,55
	P2	**35,73**	34,87	-	36,62	**35,80**	34,96	-	36,66	**35,08**	34,68	-	35,49	**35,01**	34,53	-	35,50
	P3	**35,00**	34,53	-	35,48	**35,16**	34,48	-	35,84	**35,10**	34,42	-	35,78	**34,77**	34,24	-	35,32
**HPT [°C]**	C	**45,39**	44,24	-	46,54	**44,38**	43,09	-	45,66	**44,95**	43,79	-	46,12	**44,94**	43,95	-	45,92
	P1	**44,98**	43,61	-	46,34	**45,88**	44,76	-	46,99	**45,43**	44,15	-	46,71	**44,68**	43,51	-	45,85
	P2	**45,84**	44,98	-	46,71	**45,84**	44,79	-	46,89	**45,03**	43,75	-	46,32	**45,10**	43,86	-	46,34
	P3	**44,85**	43,52	-	46,17	**46,12**	45,41	-	46,83	**45,32**	44,26	-	46,38	**45,50**	44,37	-	46,63
**SHP [VAS]**	C	**30,88**	23,35	-	40,84	**51,01**	36,47	-	71,32	**31,39**	22,05	-	44,68	**34,83**	22,99	-	52,75
	P1	**35,32**	28,25	-	44,16	**40,88**	28,70	-	58,20	**32,41**	22,15	-	47,39	**36,92**	26,20	-	52,01
	P2	**32,20**	25,44	-	40,73	**36,96**	24,96	-	54,69	**29,25**	18,56	-	46,06	**32,67**	20,54	-	51,94
	P3	**30,01**	22,35	-	40,27	**35,09**	25,76	-	47,79	**28,67**	19,37	-	42,42	**32,53**	21,33	-	49,59
**MPS [NRS]**	C	**2,92**	1,88	-	4,50	**11,55**	8,09	-	16,48	**2,76**	1,78	-	4,24	**3,24**	2,06	-	5,08
	P1	**3,41**	2,16	-	5,36	**10,94**	7,61	-	15,71	**3,52**	2,31	-	5,35	**3,73**	2,44	-	5,69
**DMA [NRS]**	C	**0,00**	0,00	-	0,00	**0,21**	0,06	-	0,49	**0,00**	0,00	-	0,00	**0,00**	0,00	-	0,01
	P1	**0,00**	0,00	-	0,00	**0,14**	0,03	-	0,32	**0,00**	0,00	-	0,00	**0,02**	-0,01	-	0,04
**flare [cm^2^]**		**0,00**	0,00	-	0,00	**41,02**	38,15	-	43,89	**0,00**	0,00	-	0,00	**0,00**	0,00	-	0,00
**area of hyperalgesia [cm^2^]**		**0,00**	0,00	-	0,00	**83,17**	76,31	-	90,02	**0,00**	0,00	-	0,00	**0,00**	0,00	-	0,00

C central test area (contact area with thermode head), DMA dynamic mechanical allodynia, HPT heat pain threshold, MPS mechanical pain sensitivity, NRS numerical rating scale, P1-3 peripheral test area, pre before conditioning stimulus (i.e. repetitive heat pain), post after conditioning heat stimuli, SHP suprathreshold rating, VAS visual analogue scale, WDT warm detection threshold.

#### Thermal testing

After heat pain conditioning, there was almost a doubling of warm detection thresholds in the conditioned primary zone (“central”) from 3.7 °C to 6.3 °C (p<0.001). A much smaller increase was encountered in the adjacent test site (“P1”; 3.5 to 4.3 °C, p<0.05), both no other ipsilateral or contralateral test area with an average pre-to-post change of +0.06°C (range: −0.33 to +0.39°C; all n.s.).

In contrast, the threshold for heat pain in the central area dropped from 45.4±0,6 °C to 44.4±0.7 °C), although this failed to reach significance (p = 0.16). There was no significant change of heat pain thresholds in any other ipsilateral or contralateral test area (on average +0.2 °C, range of changes −0.7 to +1.3°C). However, pain rating to brief suprathreshold heat pain increased from an average 31 to 51 mm on the 100 mm VAS (+65%, p<0.001), while changes in any other test area were marginal (increases on average +4 mm; range 3–6 mm, all n.s.).

#### Mechanical testing

**Figure 3:**
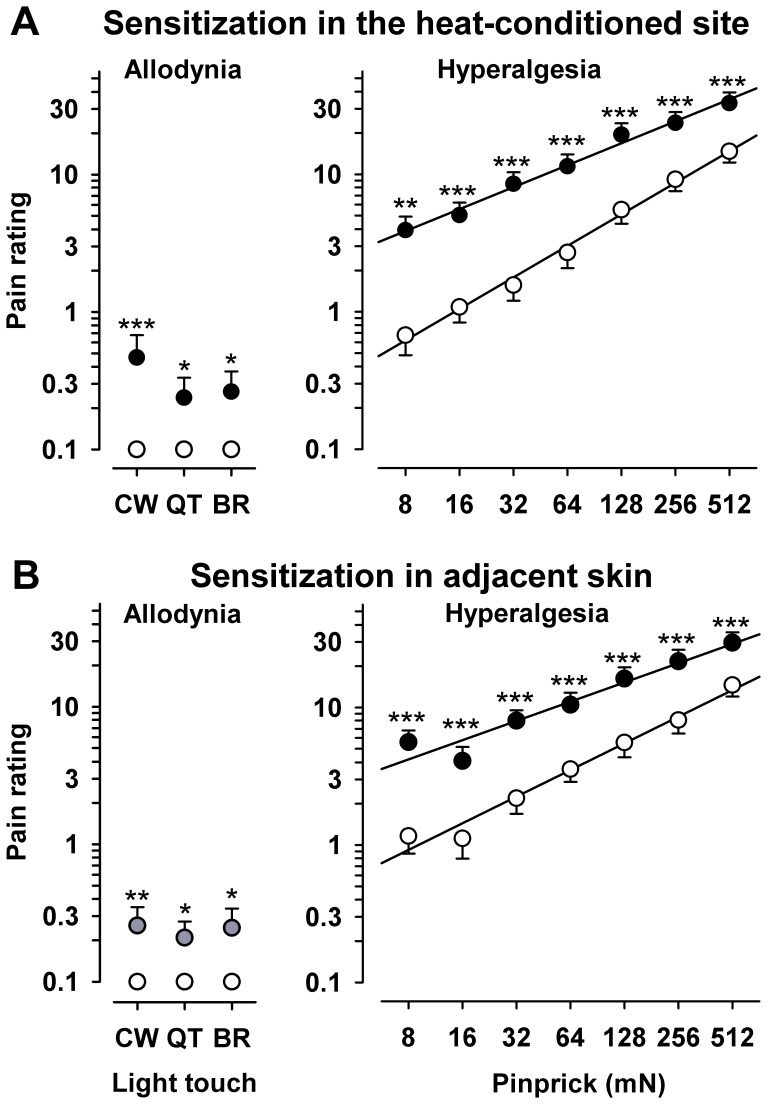
Stimulus response function for calibrated pinprick stimuli and dynamic tactile stimuli (stroking light touch) at baseline and after heat pain in A: the conditioned skin area (“central zone”) and in B: adjacent skin (“peripheral zone”). Ratings to increasing intensities of pinprick stimuli (testing for hyperalgesia) and ratings to different modalities of light touch (testing for dynamic mechanical allodynia by stroking tactile stimuli, namely by CW  = 3 mN cotton wisp, QT = 100 mN cotton wool tip, BR = 200–400 mN wide soft brush) in the central and peripheral zone. Ratings before (white) and after (black) repetitive heat pain are given on a numerical rating scale ranging from 0 (no pain) to 100 (worst imaginable pain). ** p<0.05, ** p<0.01, *** p<0.001 (t-test for repetitive heat-conditioning vs. unconditioned control).

Pinprick-evoked pain (MPS) was substantially increased by repetitive heat, approximately 4fold in the conditioned primary area (“central”) and 3.2fold in adjacent skin (“P1”; both p<<0.001), but not in the contralateral control area (increases 1.17 and 1.06fold, both n.s.). Additionally, the central and adjacent (“P1”) areas on the heat-conditioned side exhibited significant dynamic mechanical allodynia (DMA; both p<0.01), while this was not observed at the contralateral side. Detailed results of pain ratings (Figure 3) to pinprick stimuli (8 to 512 mN; right panels) and to light touch (left panels) before and after repetitive heat in the central and peripheral zone are given as stimulus-response (S/R) functions for the central area (Figure 3A) and adjacent area P1 (Figure 3B). They show that sensitization encompassed the whole range of stimulus forces (defined as a leftward shift of the S/R curve) after application of repetitive heat pain. Notably, the magnitude of hyperalgesia to pinprick stimuli was not significantly correlated to the area of secondary hyperalgesia to pinprick (r = 0.18, n.s.). Likewise, primary hyperalgesia to heat estimated either as pain rating increase during or after repetitive heating was not correlated to either area or magnitude of pinprick hyperalgesia (overall correlation r = −0.07; range: −0.27 to +0.17; all n.s.).

### Modulation of qualitative pain characteristics

**Figure 4:**
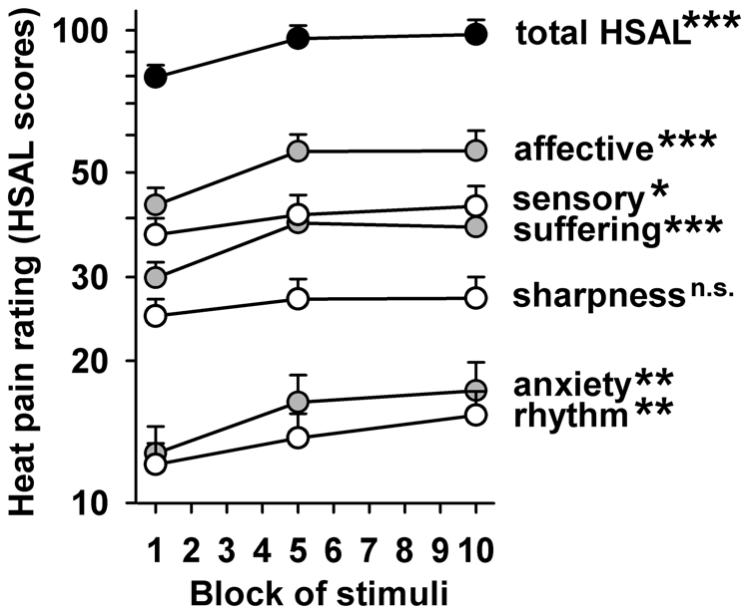
Changes of stimulus qualities by repetition of heat pain. Pain scores rated on the HSAL quality descriptor scale in the first, fifth and tenth block of heat pain conditioning. Scores generally increased upon repetition of stimulation for affective dimensions (grey circles) stronger than for sensory dimensions (open circles). HSAL compound score (total HSAL; black circles). Scores are log-scaled to allow immediate evaluation of the proportion of change across all HSAL subscores. HSAL: Hamburg Pain Adjective List. * p<0.05, ** p<0.01, *** p<0.001 (repeated measures ANOVA for factor stimulus repetition)

Analysis of pain characteristics as assessed by the HSAL list of pain descriptors yielded a substantial overall increase very similar to the increase of VAS pain ratings (+23.2%, p<0.001). Hierarchical analysis of subscales revealed that the increase in the affective pain component was more pronounced (+30%, p<0.001) than that in the sensory pain component (+15%, p<0.02) although the difference did not reach statistical significance (increase of affective vs. sensory p = 0.17). This also applied to both affective pain components, namely pain-related suffering and pain-related anxiety as well. Notably, the most purely defined sensory subscale (“pain sharpness”) only exhibited a non-significant trend (+9%, p = 0.14). All changes were already fully present at the fifth block of heat stimuli. An overview of mean values at the first, fifth and tenth block is given in Figure 4.

### Time course of primary and secondary hyperalgesia

**Figure 5:**
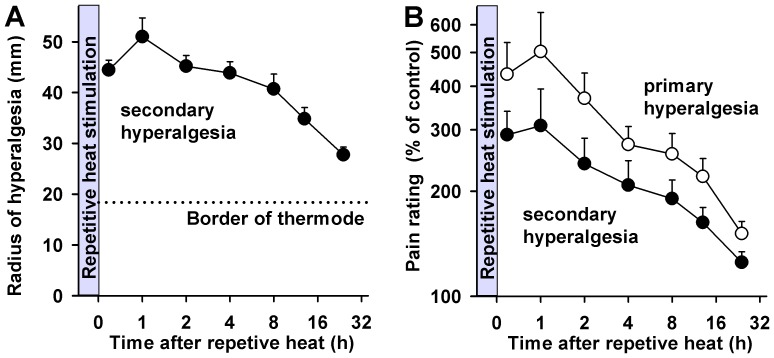
Time course of primary and secondary hyperalgesia. **A**: Time course of the area of secondary hyperalgesia over 24 h after repetitive heat revealed significant secondary hyperalgesia for at least 24 h after heat stimulation. **B**: Time course of pain ratings increase vs. the contralateral mirror image control site in the primary hyperalgesia area (open circles) and in the secondary hyperalgesia area (filled circles) showed significantly higher pain sensitivity lasting for at least 24 h after heat stimulation.

The time course of hyperalgesia parameters exhibited a maximal expression at one hour after repetitive heat pain stimulation (Fig. 5). The maximal spread of secondary hyperalgesia (Figure 5A) at that time point was 51.0 ± 3.7 mm from the thermode center (corresponding to a mean area of 81.6 cm^2^), which matched exactly the mean area in the group of 18 different subjects (compare Figure 2B). Notably, significant spread of hyperalgesia beyond the thermode area was met at any time after RHP (all at least p<0.001), and significant reduction of the secondary hyperalgesia area was only met at 8 h after RHP.

Likewise, hyperalgesia to pinprick stimulation in the primary and secondary areas peaked at one hour after RHP with a >5fold and >3fold pain increase compared to the contralateral mirror image control side (Figure 5B). Thereafter, hyperalgesia in both hyperalgesia areas slowly declined in parallel. However, significant hyperalgesia was present at any time point for primary hyperalgesia (at least p<0.001 up to 13 h after RHP, and p<0.005 at 24 h), and secondary hyperalgesia as well (at least p<0.005 up to 13 h after RHP, and p<0.05 at 24 h). Thus, hyperalgesia lasted for more than 24 h after RHP. Notably, the magnitude of pinprick hyperalgesia in the primary and secondary areas was strongly correlated up to 8 h after RHP (mean correlation: r = 0.86, p<0.001; range: 0.70–0.93) suggesting that they share a similar mechanism.

### Modulation of heat pain and heat-induced flare and secondary hyperalgesia by NSAID (acetaminophen proof-of-concept trial)

**Figure 6:**
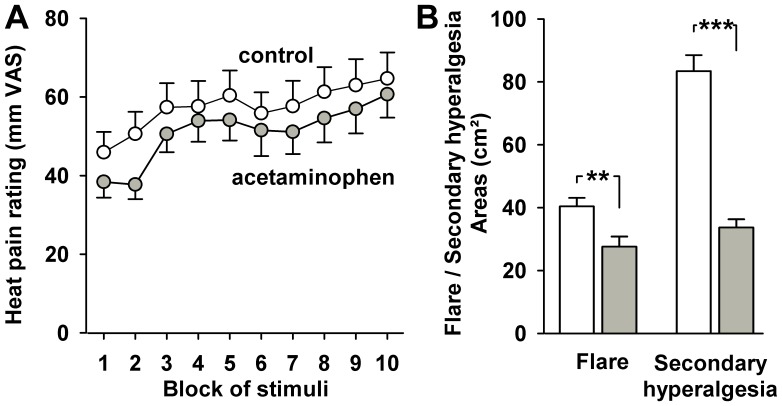
Impact of acetaminophen on heat pain upon repeated heat stimulation and areas of heat-induced flare and secondary hyperalgesia. **A**: Comparison of pain ratings in 8 healthy volunteers to repetitive heat pain stimuli after infusion of 800 mg acetaminophen (grey circles) or in the control condition (open circles; all results are given as means ± SEM). **B**: Areas of flare (left panel) and secondary hyperalgesia to punctate stimuli (mapped by 256 mN von Frey-hair; right panel) after repetitive heat in the control condition (white bars) and after i.v. acetaminophen (grey bars). ** p<0.01, *** p<0.001 (t-test for control condition vs. acetaminophen).

Ratings of repetitive heat pain in the subgroup of subjects of the acetaminophen trial (n = 8; Figure 6A) were representative of the full cohort (n = 18; for comparison see Figure 2). They increased significantly both with and without application of acetaminophen over time from the first to the tenth block (ANOVA: F = 4.733, p = 0.011). Mean pain ratings to the repetitive noxious thermal stimuli decreased slightly at any time of the conditioning heat pain protocol, and pain reduction varied from 6–26% for the ten blocks. However, in none of the blocks nor in overall pain after application of acetaminophen did the pain reduction reach statistical significance (overall pain reduction −11%, paired t-test, p = 0.41). Likewise, the intra-session development of heat hyperalgesia as calculated from the increase in heat pain ratings from the first to the tenth block of heat stimuli remained unaltered (p = 0.50). Moreover, there was no indication for a reversal of heat hyperalgesia after repetitive heat conditioning, since neither heat pain thresholds nor pain ratings to suprathreshold heat pain in the hyperalgesic area were significantly reduced (both p>0.20).

Also, flare and secondary hyperalgesia areas were representative of the full cohort. In contrast to heat pain or heat hyperalgesia, however, infusion of acetaminophen decreased the area of flare significantly by 31% from 40.4±2.7 to 27.6±3.2 cm^2^ (ANOVA; F = 15.63; p = 0.006), and the mean area of secondary hyperalgesia by 59% from 83.4±5.1 to 33.7±2.6 cm^2^ (ANOVA; F = 318.20; p<<0.001; Figure 6B). Accordingly, the reduction of the secondary hyperalgesia area was significantly more pronounced than the reduction of the flare area (p<0.02).

**Figure 7:**
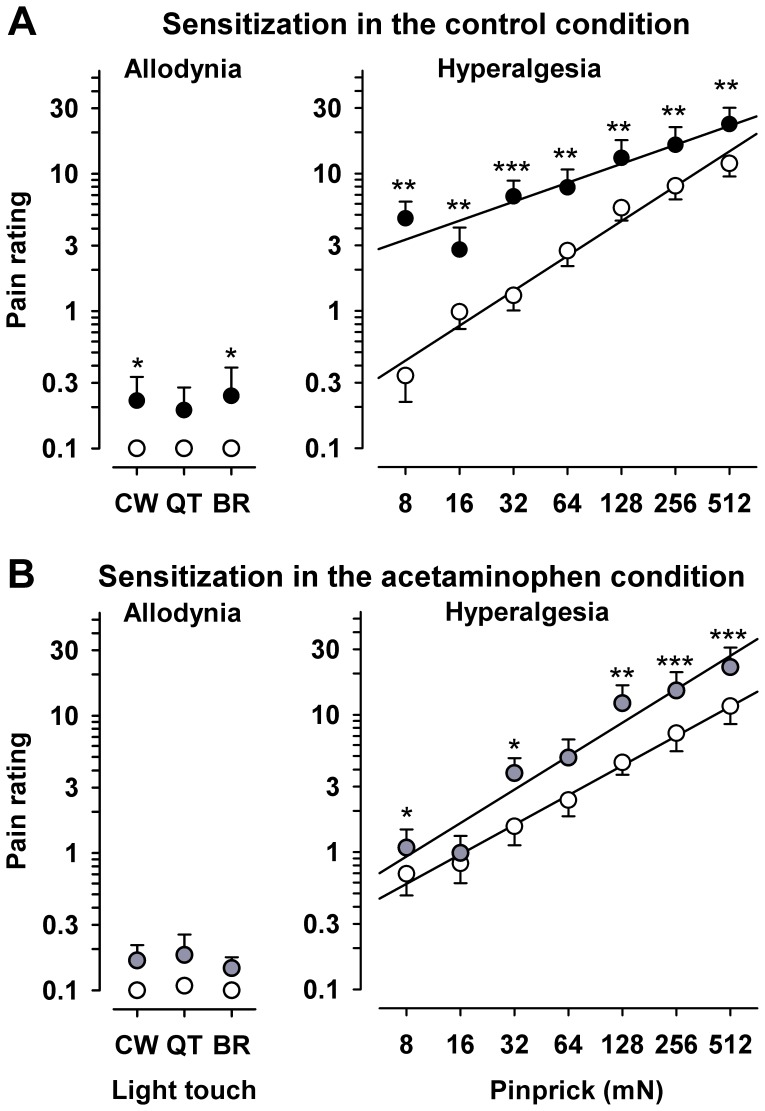
Impact of acetaminophen on pain to pinprick stimulation and magnitude of heat-induced secondary hyperalgesia and dynamic mechanical allodynia. **A**: Ratings to increasing intensities of pinprick stimuli (testing for hyperalgesia; left panel) and ratings to different modalities of light touch (testing for dynamic mechanical allodynia by stroking tactile stimuli; right panel) before (open circles) and after heat pain conditioning (black circles) in the control arm. **B**: Pain and hyperalgesia to pinprick and light touch under intravenous acetaminophen before (open circles) and after heat pain conditioning (grey circles) in the acetaminophen arm. Reduction of pinprick hyperalgesia was especially pronounced at low to moderate force pinprick stimuli. For both A and B data from the central heat-conditioned and the adjacent skin area (P1) were pooled, since both areas displayed the same magnitude of change for heat-induced hyperalgesia and allodynia as well as in acetaminophen-related reduction of hyperalgesia or allodynia. * p<0.05, ** p<0.01, *** p<0.001 (t-test for repetitive heat-conditioning vs. unconditioned control).

Potential analgesia by acetaminophen was also tested by reduction of pinprick pain. Since this was small we pooled pain ratings to pinprick in all control areas, i.e. pain rating at baseline on the test arm and pain at baseline and after heat pain conditioning in the contralateral control arm for a more stable estimate. This revealed an average reduction of pinprick pain by 8% (p>0.30 in any of the test areas). Testing hyperalgesia to pinprick and dynamic mechanical allodynia revealed that the magnitude of hyperalgesia and allodynia were similar to that of the n = 18 cohort. The magnitude did not differ in magnitude the central and in the peripheral test areas (p = 0.73 for hyperalgesia, and p = 0.75 for allodynia). Thus, both areas were thus pooled for analysis. There was a 3.23fold increase in hyperalgesia to pinprick (p<<0.001), and significant allodynia (p<0.02) after heat pain conditioning (Figure 7A).

After intravenous acetaminophen, heat pain conditioning still induced significant pinprick hyperalgesia to 1.92fold of control (p<<0.001; Figure 7B). However, this represents a significant reduction of pain in the hyperalgesic area compared to the control condition (−41%, p<0.001). Since this represents a combined analgesic and antihyperalgesic effect, we also calculated the reduction of hyperalgesia from the average shift of stimulus response functions relative to the respective contralateral control areas to subtract a potential analgesic effect, which revealed an even more prominent reduction of the hyperalgesia component (−59%, p<0.001). Notably, the reduction in pinprick hyperalgesia (magnitude) was not significantly correlated to the reduction of secondary hyperalgesia area (r = 0.18, p = 0.66). Likewise, there was significant dynamic mechanical allodynia after heat conditioning in the control arm (p = 0.012) and in the acetaminophen arm (p = 0.028; Figure 7A). Acetaminophen also reduced dynamic mechanical allodynia by approximately one third (Figure 7B), however, this failed to reach significance (p = 0.13), since allodynia was only present in approximately 40% of all tested areas (13/32).

## Discussion

The excitation of TRPV1-bearing primary afferents is essential for the induction of states of central sensitization in humans [6], [9]–[11], [26]. These afferents can be selectively excited by specific agonists like capsaicin or by noxious heat. Likewise, various models of painful heat have been used in the past to elicit central sensitization with variable success [5], [14], [27], [28]. In this investigation we modified the method of heat delivery to improve the reliability of peripheral and central sensitization and the efficacy-to-safety ratio.

After application of repetitive heat pain stimulation a strong thermal hyperalgesia was observed in the central zone below the thermode head, which is in line with previous studies showing primary heat hyperalgesia after burn injuries [4], [5]. A significant gradual increase of heat pain to repeated conditioning heat stimuli was found, which was already present in the second block of heat stimuli. However, there may be confounding factors, namely a slow increase of stimulus efficacy upon fast stimulus repetition [29], [30], but also pronounced primary afferent fatigue to repeated heat stimuli [29], [31]–[33] and some degree of centrally mediated habituation [29], [34]. In line with the assumption of primary afferent fatigue, a prominent loss of warm sensitivity was observed in the conditioned skin area. Heat hyperalgesia developed very early during and slowly mounted towards the end of the repetitive heat stimulation. However, it was seen in full bloom only later at one hour after by testing heat pain to brief heat stimuli. Since heat-sensitive nociceptors do not only sensitize, but also exhibit prominent fatigue of action potential discharge during sustained or repeated heat stimulation, which needs at least ten minutes for full recovery [29], [35], [36] we conclude that the full expression of heat sensitization only becomes apparent, when nociceptor fatigue has fully subsided and thus the sensitization prevails. A gradual development of primary afferent sensitization [37]–[39], which is limited to the stimulated site is the most likely factor contributing to the observed homotopic sensitization [40], [41],[42]. In agreement with most previous studies [4], [6], [43]–[45] no appreciable changes of heat pain thresholds or heat-induced pain ratings to suprathreshold stimuli could be observed in areas adjacent to the conditioned site and strongly argue against the induction of secondary heat hyperalgesia by our model. It is noteworthy, that ratings to suprathreshold pain ratings were more sensitive than heat pain thresholds to detect sensitization. This is consistent with findings in the quantitative sensory testing data base of the German research network on neuropathic pain that suprathreshold pain ratings exhibited less variability and higher reliability than pain thresholds in the same stimulus dimension (pinprick) [15]. There were similar findings in patients with pinprick hyperalgesia [46]. This may suggest that psychophysics of pain using the method of limits impose undue methodological constraints, like lack of sensitivity. Human psychophysical studies should thus not just rely on simple threshold assessment, but aim at understanding the processing of suprathreshold stimuli.

A significant increase in mechanical pain sensitivity and pain to light touch (dynamic mechanical allodynia) was observed in both the central and adjacent skin with similar magnitude probably resulting from a temporal and spatial summation of input from heat-sensitive nociceptors. Mapping mechanical pain sensitivity revealed large areas of secondary hyperalgesia to pinprick stimuli similar to the findings of other studies using different methods of hyperalgesia induction [4], [5], [8], [43], [47]. Both, mechanical pain sensitivity as measured by pain ratings to various pinprick intensities and the area of secondary hyperalgesia were significantly increased in our sample. Notably, they were not correlated as described previously [48] suggesting that they may represent independent aspects albeit related to the same underlying process of central sensitization (for detailed discussion see [49]) which suggests that the administration of repetitive heat pain using this paradigm is comparable to other potent inducers of experimental primary and secondary hyperalgesia, such as burn pain or capsaicin. Likewise, allodynia mediated by heterosynaptic sensitization to the central input of A-beta fibres [9] was observed in both the central and the peripheral zone following repetitive heat pain. Recently, different methods of heat-induced central sensitization were meta-analysed with two main conclusions: first, various models of noxious heat reliably induce central sensitization, and second, the most reliable results were observed with strong sustained stimulation (47°C for 7 min) [28] resulting in first degree burns, sometimes even skin blistering (second degree burn). Our model provides similar thermal stimulation (48°C for 60×6 s = 6 min plateau time) but likely more vigorous input from heat-sensitive nociceptors resulting from the phasic discharge to pulsating stimulation [50]. At the same time, intermittent cooling avoids cumulation of thermal energy to damaging levels in the tissue and ensuing burn injury [51]–[53] thus increasing the safety margin for this stimulus type. Notably, this model of repetitive heat pain stimulation induced a significantly more long-lasting type of hyperalgesia than other varieties of heat conditioning that have been published previously e.g. [51]–[53]. Rather they share the sustained time course of hyperalgesia models like high-dose intracutaneous capsaicin injection, high-frequency electrical stimulation, or experimental skin incision [51]–[53] [54], [55], suggesting that it may induce the long-term potentiation type of central sensitization. An additional advantage is the induction of a prominent and long-lasting primary heat hyperalgesia, thus allowing the parallel study of primary and secondary hyperalgesia mechanisms in the same model.

Repetitive application of noxious heat stimuli elicited a profound significant increase in all primary and secondary modalities except for pain sharpness. “Pain sharpness” reflected by ratings of adjectives like cutting, tearing, shooting, sharp, and stabbing, is the perceptual correlate of A-delta fibre activation, which is related to reflex withdrawal, while “pain rhythmicity” comprising characteristics such as nagging, hot, radiating and pulsating relates to C-fibre activation [56], [57]. The latter may directly be related to higher affective load, however, more complex central mechanisms cannot be ruled out.

As predicted from previous human studies [7], [23], the preemptive administration of acetaminophen in a proof-of-concept trial conducted in a small cohort of subjects (n = 8) changed pain ratings to repetitive heat stimuli and to painful pinpricks only marginally indicating poor analgesic efficacy. In contrast, the area of secondary pinprick hyperalgesia decreased significantly in the acetaminophen group. These findings support the notion that acetaminophen is a rather weak NSAID analgesic with some, but limited peripheral action on primary afferents. In contrast, it may be a quite powerful inhibitor of hyperalgesia of the secondary hyperalgesia type, which is related to a heterosynaptic mechanism of spinal central sensitization. This corroborates previous findings in an intracutaneous electrical hyperalgesia model [22], [23] and reports on the efficacy of acetaminophen on laser-evoked pain in panretinal photocoagulation [58] and mechanical experimental pain involving hyperalgesia to blunt pressure ^109^.

In line with previous findings the visible flare was significantly smaller than the area of secondary pinprick hyperalgesia [6], [60]. The axon reflex is of peripheral origin mediated by release of vasoactive peptides from peripheral nociceptive C fiber afferents resulting in neurogenic inflammation (capillary vasodilation visually identified as flare) [61], [62]. Consistent with previous studies acetaminophen reduced neurogenic inflammation moderately [23], [59]. This points to a minor role of the anti-inflammatory action of acetaminophen, but emphasizes its possible role as a centrally acting analgesic, more precisely as an antihyperalgesic targeting the input-driven facilitation, which is limited to gating of a specific set of primary afferents (mechanosensitive A-delta nociceptors) [6], [9]–[11], [44], [45]. This selectivity of the facilitated input is underlined by the fact, that in this study no aspect of heat pain sensitivity or heat hyperalgesia was altered. Moreover, acetaminophen exhibited no appreciable effect on any aspect of acute pain sensitivity, explaining why it has only marginal or no efficacy for ongoing nociceptive pain [63]–[65].

In contrast, there is evidence for fostering of supraspinal serotonergic pain-inhibitory pathways by acetaminophen [23], [66]–[70]. Additionally, conversion of acetaminophen to AM404, a FAAH inhibitor, prevents the breakdown of cannabinoid lipids thus enhancing cannabinoid tone and exerting an antihyperalgesic action at CB1 cannabinoid receptors at peripheral and central targets [67], [71]–[74]. This involves dampening of TRPV1 and TRPA1 action located on the central terminals of primary afferent neurons [72], [74]. This mechanisms also offer a consistent explanation for the selectivity of the acetaminophen effect, since it has been shown that descending control mechanisms may limit the expression of spinal plasticity [75]–[77]. This readily explains the rank order of efficacy that we observed: there was little or no inhibition of acute nociceptive pain (both heat and mechanical), some inhibition of the flare response, but primarily a pronounced inhibition of hyperalgesia related to central sensitization.

As shown in a previous study [40], our improved model of thermal hyperalgesia repeatedly induced a relevant intra-session peripheral and central sensitization when applied daily for more than one week. Interestingly, a relevant inter-session habituation to ratings of repetitive heat pain (RHP) was also observed. This is - at least partly – mediated centrally through the rostral part of the anterior cingulate cortex [3], [17], [78]. It is tempting to speculate that intra-session sensitization modulates inter-session habituation. Further studies using functional imaging on the spinal and cortical level, possibly using connectivity analyses, may disentangle this dual process interaction.

## Conclusion

In conclusion, our model of repetitive heat pain provides a useful method to induce pronounced peripheral sensitization (to heat) as well as centrally mediated sensitization (secondary hyperalgesia to mechanical stimuli) with a sustained time course not previously met in other heat sensitization models. Sensory and affective modalities of pain were altered significantly towards more intense ratings. This model does not only improve the efficacy/safety ratio of previous heat sensitization models. It is also relevant to further studies as it represents a convenient model for combined pharmacological testing of analgesia and anti-hyperalgesia mechanisms related to thermal and mechanical input.

## Supporting Information

Data S1 **Raw data of experiment 1 and 2.** BR 200–400 mN wide soft brush, C area of RHP application, CW 3 mN cotton wisp, DMA dynamic mechanical allodynia (pain to light touch), EXP experiment, HPT heat pain threshold, ID subject identification, MPS rating of pain to punctate stimuli (calibrated pinpricks), P1-3: skin areas adjacent to the area of RHP application, PCM acetaminophen, QT 100 mN cotton wool tip, RHP repetitive heat pain, SHP rating of suprathreshold heat pain, WDT warm detection threshold.
(XLSX)
